# Epitope mapping of CD8+ T cells on bovine leukemia virus Gag, Env and Tax protein in cattle with different bovine MHC DRB3 alleles

**DOI:** 10.1186/1742-4690-12-S1-P49

**Published:** 2015-08-28

**Authors:** Lanlan Bai, Shin-nosuke Takeshima, Ayumu Ohno, Yuki Matsumoto, Emiko Isogai, Junko Kohara, Yoko Aida

**Affiliations:** 1 Viral Infectious Diseases Unit, RIKEN, Wako, Saitama, Japan; 2 Laboratory of Animal Microbiology, Department of Microbial Biotechnology, Graduate School of Agricultural Science, Tohoku University, Sendai, Miyagi, Japan; 3 Animal Health Group, Animal Research Center, Agricultural Research Department, Hokkaido Research Organization, Local Independent Administrative Agency, Sintoku, Hokkaido, Japan

## 

The bovine leukemia virus (BLV) is the etiological agent of enzootic bovine leucosis (EBL) which is the most common neoplastic disease of cattle. Recent studies show bovine leukocyte antigen (BoLA)-DRB3 gene plays a direct role in controlling the number of proviral load in cattle and the homozygote BoLA-DRB3*1601 is associated with high proviral load. The other hand, the CD8+ cytotoxic T cell (CTL) response is an important defense against viral invasion. To find the candidate of vaccine for BLV infection we performed CD8+ T cell epitopes mapping in BLV-infected cattle with different BoLA-DRB3 alleles. In this study, 20 amino acids in length of 115 synthetic peptides were made from the sequence of Gag, Env and Tax protein of BLV, overlapping by 10 amino acids. Then total 11 CD8+ T cell epitopes were found out in BLV-infected four Japanese Black cattle with different BoLA-DRB3 alleles that are both of DRB3 *1601/1601, and DRB3 *1501/2703 and DRB3 *1501/0503 allele by WST assay. Different CD8+ T cell epitopes were recognized by cattle with different BoLA-DRB3 alleles and then dose-dependent cytotoxicity activity were measured by non-radioactive cytotoxicity assay. All of four cattle responded to gp51N11, but it has higher variability values by Wu-Kabat variability index analysis. By contrast, CD8+ T cell epitopes were determined from all of four cattle on gp30 region and they have high conservatives. gp30N16 among 4 epitopes was detected in each low proviral load cattle from susceptibility and neutrality groups. Those results were suggested that gp30 region maybe the best candidate for vaccine that can induce cell-mediated immunity against this disease.

